# Predictors of in-hospital mortality among patients with symptoms of stroke, Mashhad, Iran: an application of auto-logistic regression model

**DOI:** 10.1186/s13690-023-01084-5

**Published:** 2023-04-27

**Authors:** Ali Hadianfar, Payam Sasannezhad, Eisa Nazar, Razieh Yousefi, Mohammadtaghi Shakeri, Zahra Jafari, Soheil Hashtarkhani

**Affiliations:** 1 Student Research Committee, Mashhad University of Medical Sciences (MUMS), Mashhad, Iran; 2 Department of Neurology, Mashhad University of Medical Sciences (MUMS), Mashhad, Iran; 3 International UNESCO Center for Health-Related Basic Sciences and Human Nutrition, Mashhad University of Medical Sciences (MUMS), Mashhad, Iran; 4 Psychiatry and Behavioral Sciences Research Center, Addiction Institute, Mazandaran University of Medical Sciences, Mazandaran, Iran; 5 Department of Biostatistics, School of Public Health, Social Determinants of Health Research Center, Mashhad University of Medical Sciences (MUMS), Mashhad, Iran; 6 Clinical Research Development Unit, Ghaem Hospital, Mashhad University of Medical Sciences (MUMS), Mashhad, Iran; 7 Center for Biomedical Informatics, Department of Pediatrics, University of Tennessee Health Science Center, Memphis, USA

**Keywords:** Suspected stroke, In-hospital mortality, Emergency medical services, Auto-logistic model

## Abstract

**Background:**

Stroke is the second leading cause of death in adults worldwide. There are remarkable geographical variations in the accessibility to emergency medical services (EMS). Moreover, transport delays have been documented to affect stroke outcomes. This study aimed to examine the spatial variations in in-hospital mortality among patients with symptoms of stroke transferred by EMS, and determine its related factors using the auto-logistic regression model.

**Methods:**

In this historical cohort study, we included patients with symptoms of stroke transferred to Ghaem Hospital of Mashhad, as the referral center for stroke patients, from April 2018 to March 2019. The auto-logistic regression model was applied to examine the possible geographical variations of in-hospital mortality and its related factors. All analysis was performed using the Statistical Package for the Social Sciences (SPSS, v. 16) and R 4.0.0 software at the significance level of 0.05.

**Results:**

In this study, a total of 1,170 patients with stroke symptoms were included. The overall mortality rate in the hospital was 14.2% and there was an uneven geographical distribution. The results of auto-logistic regression model showed that in-hospital stroke mortality was associated with age (OR = 1.03, 95% CI: 1.01–1.04), accessibility rate of ambulance vehicle (OR = 0.97, 95% CI: 0.94–0.99), final stroke diagnosis (OR = 1.60, 95% CI: 1.07–2.39), triage level (OR = 2.11, 95% CI: 1.31–3.54), and length of stay (LOS) in hospital (OR = 1.02, 95% CI: 1.01–1.04).

**Conclusion:**

Our results showed considerable geographical variations in the odds of in-hospital stroke mortality in Mashhad neighborhoods. Also, the age- and sex-adjusted results highlighted the direct association between such variables as accessibility rate of an ambulance, screening time, and LOS in hospital with in-hospital stroke mortality. Thus, the prognosis of in-hospital stroke mortality could be improved by reducing delay time and increasing the EMS access rate.

## Introduction

Stroke is a major global health issue, and its burden on community health has increased over the years [1, 2]. It is the second leading cause of death and disability in adults around the world [3]. Despite its preventable nature, two-thirds of all stroke deaths occur in developing countries [4, 5]. In Iran, studies have reported the overall in-hospital mortality as 20.5% [6, 7], which is higher than developed countries [8, 9].

Identifying the risk factors of stroke mortality is one of the most important epidemiological aspects of stroke prevention that can reduce its incidence in countries [10]. One of these factors is the timely service and transfer of patients to the hospital, as stroke requires fast and advanced medical care [11]. It has been shown that for every minute of delay in the treatment of ischemic stroke, a patient typically loses approximately 1.9 million brain cells [12]. Although there are two general types of delays (pre-hospital and hospital) affecting the timing of stroke treatment, some studies have shown that pre-hospital delay (the time interval between the onset of symptoms and the patient’s arrival at the hospital) is more important and longer than hospital delay [13]. So, emergency medical services (EMS) could play an important role in the reduction of pre-hospital delays and proper management of stroke treatment [13].

Another important and influential factor in stroke management is where the patient resides. Many studies have indicated that neighborhood characteristics (such as environmental settings) and socio-economic factors could play an important role in the spread, prevention, and outcomes of the disease [14]. Concurrently, growing evidence suggests that there are remarkable geographical variations in stroke outcomes, especially post-stroke mortality [15–17]. For example, often, people living in adjacent neighborhoods are more similar regarding the risk of death than those living in a distant region [17]. Therefore, it is more appropriate to account for spatial components in health research models.

Binary logistic regression is a popular model for modeling binary outcomes. However, spatial autocorrelation is frequently present in disease occurrences and the related risk factors. To solve this issue, Besag et al. introduced an auto-logistic regression model to account for spatial structures in modeling health-related outcomes [18]. Considering the limited evidence on geographical distribution of in-hospital stroke mortality, the present study aimed to evaluate the possible geographical variations of in-hospital stroke mortality and its determinants among patients transferred by the EMS in Mashhad, Iran.

## Methods

### Study area and data sources

This study was conducted in Mashhad, the capital of Razavi Khorasan Province in Iran. Mashhad, located in northeastern Iran, is the second most populous city in Iran with an estimated population of almost 3.8 million (https://www.amar.org.ir/english/Population-and-HousingCensuses). This city has 79 ambulance vehicles in 59 stations and 25 public hospitals delivering care to patients [19]. Ghaem Hospital is a tertiary neurological referral center in the east of Iran, and all neurology emergency care is supplied at this hospital [20].

To conduct this study, we collected the data of patients with stroke symptoms transferred to Ghaem Hospital in Mashhad by the EMS between April 2018 and March 2019. This data was collected in two parts: baseline EMS information (pre-hospital) and follow-up information (screening and hospital ward information). We obtained the pre-hospital information, including delay time (the time interval between receiving an emergency call and sending an ambulance), response time (the time interval between receiving an emergency call and the arrival of an ambulance to the scene), transport time, revealed access (sum of response time and transport time), and patients’ locations from the EMS system database. Meanwhile, the follow-up information, including age, gender, screening time (hour), triage level, length of stay (LOS) in hospital, hypertension, and final stroke diagnosis based on the ICD-10 code (I63.0 to I63.9 and I69.4) was obtained from the health information system (HIS) of Ghaem Hospital. Finally, the pre-hospital data was integrated with the follow-up information using the emergency mission IDs. Furthermore, the accessibility rate of the ambulance (the number of ambulances per one million inhabitants) for each district was obtained [21]. Also, the residential addresses at admission (callers’ locations) were geocoded to latitude and longitude format using Google maps, and only patients residing inside Mashhad City were included in the study.

### Statistical analysis

The continuous and qualitative variables were presented as mean ± SD or median (IQR) and frequency (%), respectively. The comparison of quantitative variables was carried out using the independent T-Test, and the association between qualitative variables was assessed by the Chi-square test. In addition, Local Moran’s I Statistic was applied to measure the local spatial autocorrelation and clustering tendencies across neighborhoods [22]. For spatial modelling, firstly, univariate logistic regression was used and variables with *P* < 0.25 were entered in the final multiple models. Then, the auto-logistic regression model was used to determine the risk factors associated with in-hospital stroke mortality.

The auto-logistic regression model has been widely used for modeling spatially correlated binary outcomes. Compared to the ordinary logistic model, the auto-logistic model introduces a spatial autocorrelation term known as autocovariate, as shown in the following equations. Autocovariates are weighting coefficients calculated by Euclidean distance in the form of the dependent variable’s total weight.

1 $${Y}_{i}\sim Binomial \left({p}_{i}\right)$$

2 $$\mathrm{log}\left(\frac{{p}_{i}}{1-{p}_{i}}\right)={\beta }_{0}+{\beta }_{1}{X}_{1i}+\dots +{\beta }_{k}{X}_{ki}+\gamma {Autocovaraite}_{i}$$

where, *i* = *1,.., 1170* is the index of the patients; *Y*_i_ is the dependent variable corresponding to the *ith* patient, 1 if dead, 0 if alive; *p*_*i*_ is the probability of *Y*_i_ being a death case; X_1i_,..., *X*_*mi*_ are the independent variables corresponding to the *ith* patient; *β*_*0*_*,..., β*_*m*_ and γ are the regression coefficients of independent variables and *Autocovariate*_*i*_ respectively. The autocovariate variable represents spatial effects and is calculated by

3 $${Autocovariate}_{i}=\frac{\sum_{j=1}^{{n}_{i}}{w}_{ij}{y}_{j}}{\sum_{j=1}^{{n}_{i}}{w}_{ij}}$$

where ${n}_{i}$ represents the neighborhoods around the location *ith* patient, $wij$ is the spatial weight between location patient i and location patient j, equal to the inverse of Euclidean distance between *i* and *j* within the neighborhoods ($wij=1/ dij$).

Autocovariate term was calculated using the *autocov_dist* function in the *''spdep''* R package [23]. Finally, the residuals of the auto-logistic model were assessed by the Moran’s I test to ensure that the inclusion of autocovariates led to residual’s independence, and there was no spatial autocorrelation. All analyses were performed using the Statistical Package for the Social Sciences (SPSS, v. 16) and R 4.0.0 software at the significance level of 0.05.

### Ethics statement

The study protocol was approved by the Ethics Committee of the National Institute for Medical Research Development (NIMAD) (IR.NIMAD.REC.1397.078) and Mashhad University of Medical Sciences (MUMS) (Code: IR.MUMS.REC.1399.459). Data collection was accomplished by official authorization from MUMS (research project number: 981153).

## Results

### Description of the population

In this study, out of a total of 1,170 patients with stroke symptoms, 14.3% (167) patients experienced in-hospital mortality. Among them, 587 (50.1%) cases were male with a mean age of 69.92 ± 13.61 years and 584 (49.9%) cases were female with a mean age of 70.15 ± 13.93 years. Moreover, the median of LOS was 3 (IQR = 6) days, and 904 (77.2%) patients were discharged within the first week after admission. Table 1 summarizes the demographic and EMS calls characteristics of suspected stroke patients. There was no significant difference between males and females in terms of in-hospital mortality (50.1 vs. 49.9%, *P* = 0.3). Most patients were older than 60 years (75.8%), and 85.6% of deaths occurred in these patients (*P* = 0.001). The mean of delay time (*P* = 0.04) and LOS (*P* < 0.001) had a statistically significant difference in terms of death outcome. Additionally, mortality was significantly associated with triage level (*P* < 0.001) and final stroke diagnosis (*P* = 0.001). However, the mean values of variables, including response time, transport time, revealed access, accessibility rate of an ambulance, and distance to the hospital did not have a statistically significant difference between patients with and without death outcome (*P* > 0.05).

**Table 1:** Demographic and clinical characteristics of patients with symptoms of stroke

	**Total (*n* = 1170)**	**Death outcome**		***P*-value**
		**Yes (*n* = 167)**	**No (*n* = 1003)**	
**Sex, n (%)**				
Male	587 (50.1)	74 (44.3)	513 (51.1)	0.10
Female	584 (49.9)	93 (55.7)	491 (48.9)	
**Age, n (%)**				
≤ 60	283 (24.2)	24 (14.4)	259 (25.8)	0.001
> 60	888 (75.8)	143 (85.6)	745 (74.2)	
**Age, Mean ± SD**	70.0 ± 13.8	73.9 ± 13.7	69.4 ± 13.6	< 0.001
**Hypertension, n (%)**				
Yes	773 (67.5)	104 (64.2)	669 (68.1)	0.37
No	376 (32.5)	58 (35.8)	314 (32.9)	
**Residency, n (%)**				
Urban	985 (84.1)	139 (83.2)	846 (84.3)	0.74
Suburban	186 (15.9)	28 (16.8)	158 (15.7)	
**Potential accessibility rate of the ambulance (per one million inhabitants)**	27.3 ± 6.7	26.4 ± 6.7	27.5 ± 7.1	0.06
**Delay time (Sec)**	37.3 ± 29.7	42.7 ± 29.9	36.8 ± 29.7	0.04
**Response time (Min)**	9.0 ± 3.9	9.0 ± 4.1	9.0 ± 3.8	0.97
**Transport time (Min)**	21.5 ± 11.9	22.0 ± 13.9	21.5 ± 11.6	0.12
**Revealed access (Min)**	30.5 ± 13.1	31.0 ± 15.1	30.4 ± 12.8	0.61
**Screening time (hour)**	0.25 ± 0.3	0.2 ± 0.1	0.3 ± 0.3	0.04
**Distance to the hospital (km)**	5.9 ± 2.9	6.0 ± 2.9	5.9 ± 2.9	0.53
**Length of stay, median (IQR)**	3.0 (6.0)	6 (10.0)	2 (6.0)	< 0.001
**Length of stay, n (%)**				
≤ 7	904 (77.2)	94 (56.3)	810 (80.7)	< 0.001
> 7	267 (22.8)	73 (43.7)	194 (19.3)	
**Triage level**				
Levels 1 & 2	809 (69.1)	143 (85.6)	666 (66.3)	< 0.001
Levels 3 & 4	362 (30.9)	24 (14.4)	338 (33.7)	
**Final stroke diagnosis**				
Yes	299 (25.5)	60 (35.9)	239 (23.8)	0.001
No	872 (74.5)	107 (64.1)	765 (76.2)	

Results from the univariate ordinary binary logistic regression model revealed that variables, including age, final stroke diagnosis, triage level, delay time, screening time, and LOS had a significant association with in-hospital mortality in patients with symptoms of stroke. Then, the variables with a significant difference (*P* < 0.25) were entered into the final multiple logistic model (including sex, age, final stroke diagnosis, delay time, triage level, screening time, accessibility rate of ambulance, and LOS). To measure the local spatial autocorrelation, the Moran’s I test was used, the results of which showed a significant spatial autocorrelation in the multiple logistic model’s residuals. So, auto-logistic regression model, which included autocovariate term to capture the spatial autocorrelation in the model, was fitted (results in Table 2).

**Table 2:** Determining risk factors associated with in-hospital mortality in patients with symptoms of stroke using auto-logistic regression model

**Variables (Reference)**	**Multiple logistic regression**	**Multiple auto-logistic regression**
	**OR (95% CI)**	**OR (95% CI)**
**Sex (**Female**)**	Ref	
Male	1.05 (0.73–1.52)	1.05 (0.72–1.52)
**Age**	1.02 (1.01–1.04)	1.02 (1.01–1.04)
**Final stroke diagnosis (No)**		
Yes	1.61 (1.08–2.39)	1.61 (1.08–2.39)
**Triage level**		
Levels 1 & 2	Ref	
Levels 3 & 4	2.10 (1.30–3.54)	2.10 (1.30–3.53)
**Accessibility rate of ambulance (per one million residents)**	0.97 (0.94–0.99)	0.97 (0.94–0.99)
**Delay time (Sec)**	1.00 (0.99–1.01)	1.00 (0.99–1.01)
**Screening time (hour)**	0.30 (0.06–1.14)	0.30 (0.06–1.14)
**LOS**	1.02 (1.01–1.04)	1.02 (1.01–1.04)
**Autocovariate**	-	2.23 (1.36–8.14)
**AUC / AIC**	0.73/790	0.75/788

The results from the best-fitted model (multiple auto-logistic regression) showed that variables age (OR = 1.02, 95% CI: 1.01–1.04), final stroke diagnosis (OR = 1.60, 95% CI: 1.07–2.39), triage level (OR = 2.11, 95% CI: 1.31–3.54), LOS (OR = 1.02, 95% CI: 1.01–1.04) and accessibility rate of ambulance (OR = 0.97, 95% CI: 0.94–0.99) had a statistically significant association with in-hospital mortality among patients with symptoms of stroke (*P* < 0.05). After adjusting for the effect of other variables in the model, age and LOS were found to be positively associated with in-hospital mortality, with the odds of in-hospital mortality increasing by 2 percent per unit increase in age and LOS. Also, the accessibility rate of ambulance is negatively associated with in-hospital mortality, and the odds of in‐hospital mortality decreased by 3 percent per each unit increase in the accessibility rate of ambulance (per one million residents). In addition, after controlling the effect of other variables, the odds of in‐hospital mortality in patients with a final stroke diagnosis was 1.60 times compared to suspected stroke patients. In other words, the odds of in‐hospital mortality in patients with a final stroke diagnosis was 60 percent more than in suspected stroke patients. Patients with triage at the level of 3 & 4 had 2.11 times higher odds of in-hospital mortality than those with a triage level of 1 & 2. Other variables did not show a statistically significant association with in‐hospital mortality among patients with symptoms of stroke (*P* > 0.05) (Table 2).

The Moran’s I statistic results showed no spatial autocorrelation in the residuals from the auto-logistic regression model. The auto-logistic regression model is preferred over the ordinary logistic regression model because it could cover spatial autocorrelations in the in-hospital mortality of suspected stroke patients. Then, and receiver operating characteristic curve (ROC) analyses were used for model evaluation. The area under the curve (AUC) was obtained as 75.79% (65.0–73.5%), indicating the selected model’s adequate predictive ability. Furthermore, based on Akaike Information Criterion (AIC), auto-logistic model had better performance compared to the ordinary logistic regression model (AIC = 788 vs. AIC = 790).

**Fig. 1:**
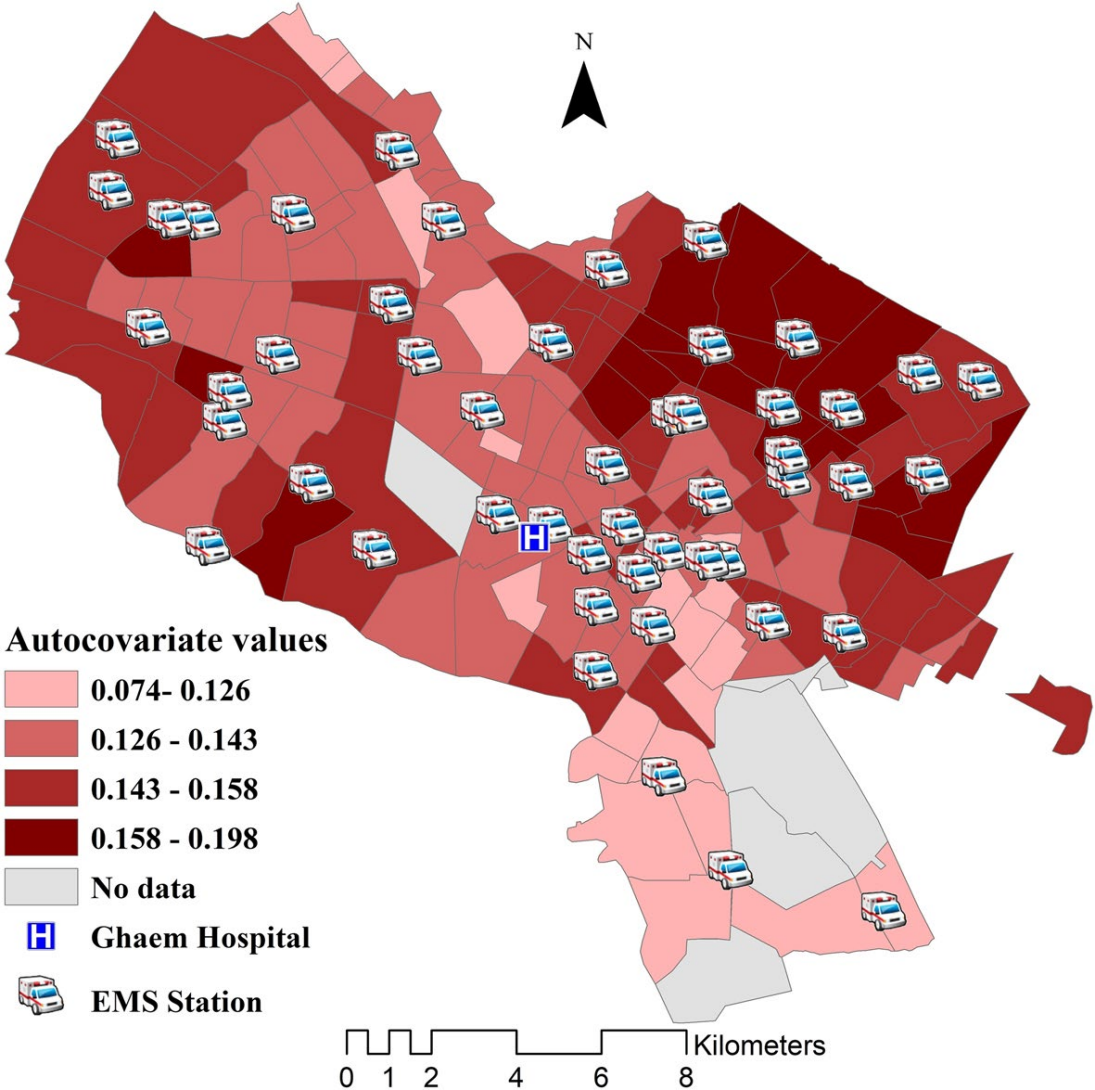
The spatial distribution of the autocovariate variable in the neighborhood, Mashhad

Figure 1 maps the estimated values of the autocovariate variable introduced in Eq. (3). The larger values of the autocovariate variable indicate neighborhoods with higher odds of in-hospital stroke mortality. We witnessed a remarkable geographical variation in in-hospital stroke mortality in Mashhad neighborhoods, in a way that some neighborhoods in the suburban areas and northeastern parts of Mashhad had a higher odds of in-hospital stroke mortality. Also, in the northeastern part of the study area, the difference was more notable due to the socio-economic deprivations, such as having low-income occupations, lower availability, quality of primary care, and less education. Also, some other neighborhoods had high odds of in-hospital mortality, which could be due to having more pensioners or pre-retirees and an older population.

## Discussion

This study investigated possible disparities in in-hospital mortality and its determinants among patients with symptoms of stroke transferred by EMS to Ghaem Hospital in Mashhad, Iran. According to our results, there was a noticeable spatial variation in in-hospital mortality among patients with symptoms of stroke in Mashhad, Iran.

The overall in-hospital stroke mortality rate in this study was obtained as 14.3%, which is higher than some other studies [24–26]. The possible reason could be related to the fact that most stroke patients (75.8%) in our study were over 60 years old. However, the mortality rate in our study was lower than some other studies [8, 9, 27, 28].

In our study, there was a significant spatial autocorrelation between observations according to Moran’s I test. To tackle this issue, the auto-logistic regression model was applied, because it is necessary to consider this spatial autocorrelation in modeling. The results from the auto-logistic regression model showed that variables, including age, LOS, final stroke diagnosis, and triage level had a significant association with in-hospital stroke mortality. Furthermore, the autocovariate term was significant; so, the odds of in-hospital stroke mortality were higher in the suburbs and northeastern areas. Several arguments may justify these findings. For example, northeastern neighborhoods of Mashhad have a very high population density but low health facilities. In other words, in Mashhad, the focus of health services is mostly on the city’s central neighborhoods. As a result, central areas of the city have higher access to health services than the suburbs [27, 28]. Therefore, health policymakers should pay attention to inequalities in healthcare resources’ spatial distribution. Another reason for the higher probability of mortality in suburbs and northeastern areas is the low socio-economic status. Also, there are clusters of urban poverty in the eastern part and marginal neighborhoods in Mashhad [29].

Our findings indicated that age is an important independent factor of death, so that each additional year is associated with a 3% relative increase in in-hospital stroke mortality. This result is in line with other studies that indicated a positive relationship between age and in-hospital stroke mortality [30–32].

In our study, the median LOS was 6.0 days, and 22.8% of patients were hospitalized for more than 1 week. The model results indicated that longer LOS increases the odds of in-hospital mortality, so that for each day of increase in LOS, the odds of in-hospital mortality increase by 2%. Previous studies showed a strong positive relationship between LOS and in-hospital stroke mortality [6, 26, 33].

The mean accessibility rate of an ambulance in Mashhad city was 27 (per one million inhabitants) in 2018 [21]. Our findings indicated that the accessibility rate of an ambulance was negatively associated with in-hospital mortality. With an increase in the accessibility rate of an ambulance, the odds of in-hospital stroke mortality decrease. Previous studies showed that more access to EMS increased the likelihood of therapy with rt-PA and decreased visiting time by an emergency physician, time to CT, and visiting time by a neurologist [34–36]. Our results also revealed that the access rate to EMS in the suburbs was lower than in the central urban areas due to the huge aggregation of EMS stations in central areas. So, stroke patients in suburban areas were less likely to receive timely therapy and had higher in-hospital mortality [37]. Therefore, health policymakers should pay attention to EMS stations’ balanced distribution, especially in areas with high gaps and low EMS access rates.

In this study, we only integrated the data obtained from the EMS system database of the City Emergency Management Center with patient medical records in the Ghaem Hospital. However, this study had several limitations. First, patients who came to the hospital by personal vehicle were not considered in this study, because the crucial purpose of our study was to investigate the performance of EMS in the transfer of stroke patients from different areas of Mashhad to Ghaem Hospital and to evaluate its impact on the occurrence of in-hospital mortality. Second, our study was based on data acquired in a year, which may differ from in-hospital stroke mortality patterns in multi-year data-based studies. Third, since registry-based data was used in this study, variables such as stroke type (hemorrhage or infarct), stroke severity, hospital discharge policy, comorbidities, etc. were not entirely recorded and many cases were registered as unspecified.

## Conclusion

Our findings revealed a relatively noticeable geographical variation in the odds of in-hospital stroke mortality in Mashhad, Iran. Exploring areas with higher odds of mortality could help health policymakers to design better policies and allocate resources. As a result, this could improve the performance of EMS and reduce in-hospital stroke mortality. Finally, our results also indicated that the variables, including age, screening time, accessibility rate of an ambulance, and LOS had a significant association with in-hospital stroke mortality.

## Data Availability

The datasets generated and/or analyzed during the current study are not publicly available due [their containing information] but are available from the corresponding author on reasonable request.
